# Toward a Microencapsulated 3D hiPSC-Derived *in vitro* Cardiac Microtissue for Recapitulation of Human Heart Microenvironment Features

**DOI:** 10.3389/fbioe.2020.580744

**Published:** 2020-11-05

**Authors:** Bernardo Abecasis, Pedro G.M. Canhão, Henrique V. Almeida, Tomás Calmeiro, Elvira Fortunato, Patrícia Gomes-Alves, Margarida Serra, Paula M. Alves

**Affiliations:** ^1^iBET, Instituto de Biologia Experimental e Tecnológica, Oeiras, Portugal; ^2^Instituto de Tecnologia Química e Biológica António Xavier, Universidade Nova de Lisboa, Oeiras, Portugal; ^3^CENIMAT| i3N, Departamento de Ciência dos Materiais, Faculdade de Ciências e Tecnologia, Universidade NOVA de Lisboa, Caparica, Portugal

**Keywords:** hiPSC, cardiomyocytes, endothelial cells, fibroblasts, engineered cardiac tissues, microencapsulation, 3D culture

## Abstract

The combination of cardiomyocytes (CM) and non-myocyte cardiac populations, such as endothelial cells (EC), and mesenchymal cells (MC), has been shown to be critical for recapitulation of the human heart tissue for *in vitro* cell-based modeling. However, most of the current engineered cardiac microtissues still rely on either (i) murine/human limited primary cell sources, (ii) animal-derived and undefined hydrogels/matrices with batch-to-batch variability, or (iii) culture systems with low compliance with pharmacological high-throughput screenings. In this work, we explored a culture platform based on alginate microencapsulation and suspension culture systems to develop three-dimensional (3D) human cardiac microtissues, which entails the co-culture of human induced pluripotent stem cell (hiPSC) cardiac derivatives including aggregates of hiPSC–CM and single cells of hiPSC–derived EC and MC (hiPSC–EC+MC). We demonstrate that the 3D human cardiac microtissues can be cultured for 15 days in dynamic conditions while maintaining the viability and phenotype of all cell populations. Noteworthy, we show that hiPSC–EC+MC survival was promoted by the co-culture with hiPSC–CM as compared to the control single-cell culture. Additionally, the presence of the hiPSC–EC+MC induced changes in the physical properties of the biomaterial, as observed by an increase in the elastic modulus of the cardiac microtissue when compared to the hiPSC–CM control culture. Detailed characterization of the 3D cardiac microtissues revealed that the crosstalk between hiPSC–CM, hiPSC–EC+MC, and extracellular matrix induced the maturation of hiPSC–CM. The cardiac microtissues displayed functional calcium signaling and respond to known cardiotoxins in a dose-dependent manner. This study is a step forward on the development of novel 3D cardiac microtissues that recapitulate features of the human cardiac microenvironment and is compliant with the larger numbers needed in preclinical research for toxicity assessment and disease modeling.

## Introduction

The development of reliable human *in vitro* cardiac tissues that better mimic the physiology of the human heart is critical to bridge the gap between the animal models currently used in preclinical research and the human clinical setting. Human induced pluripotent stem cells (hiPSCs) have emerged as a relevant tool for human *in vitro* cardiac cell-based modeling, mainly due to (i) the human origin, (ii) the unlimited proliferative capacity, (iii) the ability to differentiate into different cardiac cell lineages, and (iv) the potential for personalized (patient-specific) medicine.

Recapitulation of the human heart microenvironment in physiological or pathophysiological context for *in vitro* cell-based modeling requires the utilization of the different cellular populations that compose the human heart, namely, cardiomyocytes (CM), endothelial cells (EC), and mesenchymal cells (MC; which comprise mainly cardiac fibroblasts, pericytes, and smooth muscle cells). Although there is a general consensus that CM constitute approximately 30% of the mammalian heart, estimated frequencies of the other populations have been involved in some controversy (Zhou and Pu, 2016). One of the reasons for this controversy is not only the interspecies or intertissue variations but also the dynamic turnover of the human heart during aging (Zhou and Pu, 2016). For example, in the postnatal period, CM represent the majority of the cardiac cells (66%), as opposed to the 30% in the adult mammalian heart (Bergmann et al., 2015). Another reason for this controversy is that divergent results have been obtained using different analytical tools. Nevertheless, the proximity between these different cell populations enables for intercellular communication mediated by paracrine factors, cell–cell interactions, and/or extracellular matrix (ECM) deposition (Kofron and Mende, 2017). Thus, the derivation of these cellular components from hiPSC has been pursued by our group and others. Nevertheless, current protocols for generation of CM from hiPSC (hiPSC–CM) result in cells with an immature phenotype, showing metabolic, structural, and functional characteristics that more closely resemble fetal CM rather than adult CM (Correia et al., 2017). Studies with three-dimensional (3D) hiPSC–derived engineered cardiac tissues have shown a positive impact of cardiac non-myocyte populations (EC and MC) on hiPSC–CM structural and functional maturation (Burridge et al., 2014; Masumoto et al., 2016; Ravenscroft et al., 2016; Giacomelli et al., 2017, 2020). However, most of these studies have either used simplistic 3D spheroid configurations (Ravenscroft et al., 2016; Giacomelli et al., 2017, 2020) and/or hydrogels composed of undefined matrix components, such as Matrigel^TM^ (Thavandiran et al., 2013; Burridge et al., 2014; Masumoto et al., 2016; Nakane et al., 2017). Moreover, the current methodologies for production of such cardiac tissue models are still limited due to (i) low throughput of aggregation protocols dependent of 96-well plate forced aggregation (Ravenscroft et al., 2016; Giacomelli et al., 2017, 2020); (ii) dependence on microfabrication tools (Thavandiran et al., 2013; Nakane et al., 2017); and (iii) low-throughput hydrogel encapsulation techniques (Burridge et al., 2014; Masumoto et al., 2016). Thus, further effort should be performed to develop protocols that enable the scalable production of a biologically relevant hiPSC-derived cardiac tissue model in defined conditions for further implementation of large pharmacological screenings.

In this work, we aim to recapitulate human heart microenvironment features through the design of a 3D human cardiac microtissue by exploring a culture platform based on xeno-free alginate microencapsulation of hiPSC–CM aggregates and single cells of hiPSC–derived EC and MC (hiPSC–EC+MC). We characterize the 3D hiPSC–derived cardiac microtissue at the phenotypic, structural, and functional levels and performed a proof-of-concept toxicological analysis.

## Materials and Methods

### hiPSC Culture and Differentiation Into Cardiovascular Lineages

#### hiPSC Expansion

In this study, hiPSC line DF19-9-11T.H from WiCell was used. hiPSCs were cultured on Matrigel^®^ (Corning)–coated plates in mTESR1 medium (STEMCELL Technologies, hereafter designated as expansion medium) at 37°C, in a humidified atmosphere of 5% CO_2_ (vol/vol) in air. Cells were routinely subcultured when reaching 80% confluence using Versene (Thermo Fisher Scientific) as described by our group (Correia et al., 2016).

#### hiPSC–CM Differentiation

Differentiation of hiPSCs into CM was performed using the 3D protocol described before by our group (Correia et al., 2018). Briefly, hiPSCs were cultured as monolayers as described in section “*hiPSC Expansion*,” and differentiation into CM was initiated when cell confluence reached 80 to 90%. At this timepoint (day 0), expansion medium was replaced by RPMI medium (Thermo Fisher Scientific) supplemented with B27 without insulin (RPMI/B27, Thermo Fisher Scientific), 12 μM CHIR99021 (Tocris), 80 ng/mL activin A (PeproTech), and 50 μg/mL ascorbic acid (Sigma–Aldrich). After 24 h, the medium was replaced by RPMI/B27 supplemented with 5 μM IWR-1 (Sigma–Aldrich) and 50 μg/mL ascorbic acid (Sigma–Aldrich). At day 3 (72 h after differentiation induction), medium was exchanged for RPMI/B27 supplemented with 5 μM IWR-1. At day 7, cell monolayers were dissociated by incubation with TrypLE Select (Thermo Fisher Scientific) for 5 min at 37°C, followed by addition of culture medium (RPMI/B27) and centrifugation at 220 × *g* for 5 min at room temperature (RT, 18°C–20°C). Single cells were then inoculated in AggreWell^TM^400Ex plates (Stem Cell Technologies) at 1,500 cells/microwell, centrifuged at 100 × *g* for 3 min at RT, and cultured in RPMI/B27 medium. 2 days after seeding, the generated cell aggregates were transferred to shake flasks (i.e., Erlenmeyer) and cultured in RPMI/B27 medium at an agitation rate of 90 rpm for additional 6 days (total culture time, 15 days). Medium was replaced every 2 days. During differentiation period, cells were cultured at 37°C, in a humidified atmosphere of 5% CO_2_ (vol/vol) in air. At day 15, hiPSC–CM aggregates were dissociated, counted by trypan blue exclusion assay (see section “*Cell Counting, Viability, and Metabolic Activity”*), and characterized by flow cytometry (see section “*Flow Cytometry of hiPSC–CM”*). These hiPSC–CM aggregates were then encapsulated in alginate as described below (see section “*3D hiPSC–Derived Cardiac Microtissue Culture”*).

#### hiPSC–EC+MC Differentiation

Human iPSCs were propagated as described in section “*hiPSC expansion*” and endothelial/mesenchymal differentiation was induced according to the protocol published by Giacomelli et al., 2017. Briefly, hiPSCs were seeded at 12.5 × 10^3^ cells/cm^2^ on the day before starting the differentiation (day -1). At day 0, cardiac mesoderm was induced by changing the medium to APEL-LI medium (Thermo Fisher Scientific), supplemented with a mixture of cytokines (20 ng/mL BMP4, PeproTech; 20 ng/mL activin A, PeproTech; and 1.5 μM GSK3 inhibitor CHIR99021, Tocris). At day 3, cytokines were removed, and vascular endothelial growth factor (VEGF; 50 ng/mL, PeproTech) was added. APEL-LI medium supplemented with VEGF was refreshed every 3 days until day 10. During differentiation cells were cultured at 37°C, in a humidified atmosphere of 5% CO_2_ (vol/vol) in air. At day 10, hiPSC–EC+MC were harvested using TrypLE Select, counted by trypan blue exclusion assay (see section “*Cell Counting, Viability, and Metabolic Activity”*), and characterized by flow cytometry (see section “*Flow Cytometry of hiPSC–CM”*). These cells were then encapsulated in alginate as described below (see section “*3D hiPSC–Derived Cardiac Microtissue Culture”*).

### 3D hiPSC–Derived Cardiac Microtissue Culture

The 3D hiPSC–derived cardiac microtissues were developed using microencapsulation technology. Alginate microencapsulation of cells/aggregates was performed as described previously (Rebelo et al., 2015, 2018; Estrada et al., 2016). More specifically, hiPSC–CM aggregates [total of 1.2 × 10^4^ aggregates corresponding to approximately 5 × 10^6^ cells, counted by trypan blue exclusion assay (see section “*Cell Counting, Viability, and Metabolic Activity”*)], were collected from suspension cultures and mixed with single cells of hiPSC–EC+MC (10 × 10^6^ cells, harvested from adherent cultures and counted by trypan blue exclusion method) for a ratio of 1:2 (hiPSC–CM:hiPSC–EC+MC). The suspension of hiPSC–CM aggregates and hiPSC–EC+MC single cells was centrifuged at 300 × *g*, for 5 min at RT, and resuspended in 1 mL of 1.1% (wt/vol) of 1:1 mixture of Ultrapure MVG alginate (NovaMatrix, Pronova Biomedical) and NOVATACH MVG GRGDSP peptide-coupled alginate (NovaMatrix, Pronova Biomedical) dissolved in NaCl 0.9% (wt/vol) solution. Cell microencapsulation was performed using an electrostatic bead generator (VarV1, Nisco), to produce beads of approximately 1,500 μm in diameter. The alginate droplets were cross-linked in a 100 mM CaCl_2_/10 mM HEPES (pH 7.4) solution for 7 min, washed three times in a 0.9% (wt/vol) NaCl solution and then equilibrated in culture medium [1:1 mixture of RPMI medium supplemented with B27 without insulin and Endothelial Cell Growth Medium 2 (Promocell)]. The microencapsulated microtissues were then transferred to shake flasks (50 mL of culture, approximately 14 capsules/mL) and cultured in suspension at 90 rpm in a humidified incubator with 5% (vol/vol) CO_2_ in air at 37°C for 15 days. Medium exchange was performed every 2 to 3 days (three times a week). Monocultures of hiPSC–CM (5 × 10^6^ cell/mL of alginate) and co-cultures of hiPSC–EC+MC (10 × 10^6^ cell/mL of alginate) were also microencapsulated and used as controls.

### Cell Culture Characterization

#### Flow Cytometry of hiPSC–CM

Human iPSC–CM aggregates were harvested from culture and dissociated with TypLE^TM^ Select for 5 min at 37°C with agitation. Afterward, single cells were washed twice with Dulbecco’s phosphate-buffered saline (DPBS) (Thermo Fisher Scientific), and 5 × 10^5^ cells were incubated in the dark with one of the following conjugated antibodies for 1 h at 4°C: SIRPα/β (CD172a/b-PE, BioLegend, diluted 1:20 in DPBS), VCAM (CD106-PE, BD Biosciences, diluted 1:5 in DPBS), or isotype control immunoglobulin G1 (IgG1),κ-PE (BD Biosciences, diluted 1:5 in DPBS). For detection of intracellular marker (Troponin T, Thermo Fisher Scientific), cells were fixed and permeabilized with Inside Stain Kit (Miltenyi Biotec) according to the manufacturer’s instructions. Cells were incubated with primary antibody (diluted 1:200 in InsidePerm) for 10 min at RT, washed with InsidePerm, and incubated with secondary antibody anti–mouse IgG Alexa Fluor 488 (diluted 1:200 in InsidePerm) for 10 min at RT. Cells were washed with InsidePerm and analyzed by a CyFlow^®^ space (Partec GmbH). Ten thousand events were analyzed per sample.

#### Flow Cytometry of hiPSC–EC+MC

Human iPSC–EC+MC were harvested from culture by dissociation with TypLE^TM^ Select for 5 min at 37°C. Afterward, single cells were washed twice with DPBS, and 5 × 10^5^ cells were processed as described in section “*Flow Cytometry of hiPSC–CM*.” Primary antibodies used were CD31 (Agilent, diluted 1:50 in DPBS), VE-cadherin (R&D Systems, diluted 1:13 in DPBS), vimentin (Abcam, diluted 1:100 in InsidePerm), or α-smooth muscle actin (α-SMA; Agilent, diluted 1:100 in InsidePerm). Secondary antibody used was anti–mouse IgG Alexa Fluor 488 (diluted 1:200 in InsidePerm). Cells were analyzed by a CyFlow^®^ space (Partec GmbH). Ten thousand events were analyzed per sample.

#### Cell Counting, Viability, and Metabolic Activity

Viable cells were quantified by trypan blue exclusion, as described elsewhere (Abecasis et al., 2017). For viability assessment, the enzyme substrate fluorescein diacetate (FDA, Sigma–Aldrich) and the DNA dye propidium iodide (PI, Sigma–Aldrich) were used (Serra et al., 2011; Silva et al., 2015). In this method, direct staining of the live aggregates was performed followed by observation at the fluorescence microscope (DMI6000, Leica, Wetzlar, Germany), as described elsewhere (Serra et al., 2011). Cells that accumulated the metabolized product of FDA were considered live, and cells stained with PI were considered dead.

For evaluation of metabolic activity, the reduction capacity of the cultures was measured by PrestoBlue^®^ Viability Reagent reduction assay (Life Technologies), according to the manufacturer’s instruction. PrestoBlue^®^ reagent is reduced by viable cells and becomes highly fluorescent. This color change can be detected using fluorescence measurements. Samples of 2 to 5 capsules were taken from suspension agitated cultures into a 96-well plate. The microencapsulated cells were incubated with PrestoBlue^®^ reagent for 3 h at 37°C. After this period, the supernatant was collected, and the fluorescence was read at 560-nm excitation and 590-nm emission in the microplate reader Infinite^®^200 PRO (NanoQuant, Tecan Trading AG).

#### Gene Expression Analysis

Alginate microcapsules of cardiac microtissues were dissolved with a chelating solution (sodium citrate 50 mM, sodium chloride 100 mM) and centrifuged at 300 × *g* for 5 min at RT. Pellets were snap-frozen and kept at -80°C until RNA isolation. Total RNA was isolated with the High Pure RNA Isolation Kit (Roche). The RNA was quantified, and purity checked by Nanodrop 2000c (Thermo Scientific). RNA reverse transcription to double-stranded cDNA was performed with the Transcriptor High Fidelity cDNA Synthesis Kit (Roche). Real-time quantitative polymerase chain reaction (RT-qPCR) was conducted with the LightCycler 480 Probes Master system (Roche) using TaqMan Gene Expression Assays (Thermo Fisher Scientific). The performed cycles were as follows: preincubation at 95°C for 10 min, 45 cycles of amplification with denaturation at 95°C for 15 s, and annealing at 60°C for 1 min; extension at 72°C for 5 min. Threshold cycles (Ct) were automatically determined by the LightCycler 480 Software (Roche). All data were analyzed using the 2^–Δ^ ^Δ^ ^*Ct*^ method for relative gene expression analysis (Livak and Schmittgen, 2001). Changes in gene expression were normalized using the Ct geometric mean of housekeeping genes *RPLP0* and *GAPDH*.

#### Immunofluorescence Microscopy

##### Cryosectioning

Microencapsulated cells were collected from culture and fixed in 4% (wt/vol) formaldehyde with 4% (wt/vol) sucrose in phosphate-buffered saline (PBS) for 20 min at RT. For cryosectioning preparation, samples were dehydrated in 30% (wt/vol) sucrose overnight at 4°C, embedded in Tissue-Tek^®^ O.C.T. (Sakura) and frozen at -80°C. The frozen samples were sliced with a thickness of 10 μm in a cryostat (Cryostat CM 3050 S, Leica). The cryosections were permeabilized for 10 min with 0.1% (vol/vol) Triton X- 100 (Sigma–Aldrich) and blocked with 0.2% (wt/vol) fish-skin gelatin (FSG; Sigma–Aldrich) in PBS for 30 min. Primary and secondary antibodies were prepared in 0.125% (wt/vol) of FSG in PBS and incubated for 2 h. The primary antibodies used were as follows: cardiac troponin T (cTnT, Thermo Fisher Scientific, 1:200), sarcomeric α-actinin (Sigma–Aldrich, 1:200), collagen I (Abcam, 1:100), and collagen IV (Abcam, 1:100). Secondary antibodies used were as follows: anti–mouse IgG Alexa Fluor 594, anti–rabbit IgG Alexa Fluor 594, anti–mouse IgG Alexa Fluor 488, anti–rabbit IgG Alexa Fluor 488, and anti–mouse IgG Alexa Fluor 594 (all from Thermo Fisher Scientific, 1:500). The samples were mounted in Prolong^®^ Gold reagent containing DAPI (Life Technologies). Samples were visualized using a confocal fluorescence microscope (SP5, Leica).

##### Whole mount

Microencapsulated cells were collected from culture and fixed in 4% (wt/vol) formaldehyde with 4% (wt/vol) sucrose in PBS for 20 min at RT. Samples were permeabilized and blocked with 1% (wt/vol) Triton X-100 solution/0.2% FSG for 2 h at RT and subsequently incubated overnight at RT with primary antibodies (CD31, Agilent; vimentin, Abcam) diluted in 0.1% (wt/vol) TX-100 and 0.125% (wt/vol) FSG. Samples were then washed three times with DPBS and incubated with secondary antibodies (AlexaFluor 488 goat anti–mouse IgG, AlexaFluor 549 goat anti–rabbit IgG, Thermo Fisher Scientific) diluted in 0.125% (wt/vol) FSG, for 5 h at RT. After three washes with DPBS, cell nuclei were counterstained with DRAQ5 (Thermo Fisher Scientific). Samples were visualized using light-sheet fluorescence microscopy (LSFM) or multiphoton immunofluorescence microscopy (IFM) as described previously (Estrada et al., 2016).

#### Transmission Electron Microscopy

Microencapsulated cells were fixed in 4% (wt/vol) paraformaldehyde with 4% (wt/vol) sucrose in DPBS for 20 min at RT. Fixed samples were washed twice with DPBS and stored at 4°C. Second fixation was performed using 2% (vol/vol) formaldehyde (EMS) and 2.5% (vol/vol) glutaraldehyde (Polysciences) in 0.1 M phosphate buffer (PB), for 1 h at 4°C. Cells were washed with PB and embedded in 2% low melting point agarose (OmniPur) for further processing. Postfixation was performed with 1% (wt/vol) osmium tetroxide (EMS) in 0.1 M PB for 30 min on ice in the dark. After two washes with 0.1 M PB and two washes with water, samples were incubated with 1% aqueous tannic acid (wt/vol; EMS) for 20 min on ice. After five washes with water, samples were contrasted with 0.5% aqueous uranyl acetate (wt/vol), 1 h on ice in the dark. Dehydration was performed using a graded series of ethanol (30, 50, 75, 90, and 100%), and samples were embedded in Embed-812 epoxy resin (EMS). Ultrathin sections were cut on a Leica UC7 ultramicrotome. Sections were collected on grids coated with 1% (wt/vol) formvar (Agar Scientific) in chloroform (VWR) and stained with 1% (wt/vol) uranyl acetate and Reynolds lead citrate, for 5 min each. Images were taken on a Hitachi H-7650 at 100 keV equipped with a XR41M mid mount AMT digital camera.

#### Calcium Imaging

Calcium imaging was performed using the Fluo-4 Direct^TM^ Calcium Assay Kit (Thermo Fisher Scientific) according to the manufacturer’s instructions. Briefly, microencapsulated cells were transferred onto four-well μslides (Ibidi). The samples were loaded with the calcium indicator dye Fluo-4 (diluted 1:2 in culture medium) for 45 min at 37°C plus 15 min at RT and washed twice with culture media. Analysis was performed under temperature control (37°C), and spontaneous calcium activity was recorded. Cells were analyzed alone or exposed to norepinephrine (60 μM, norepinephrine bitartrate, Sigma–Aldrich). Alginate microcapsules were imaged using a spinning disk confocal microscope (Revolution XD, Andor), and 30-s videos were acquired using Micro-Manager 1.4 software. Fluorescence videos were analyzed by manually selecting each individual aggregate within the alginate microcapsule using ImageJ open source software (Rasband, WS, ImageJ, United States National Institutes of Health, Bethesda, MD, United States, http://imagej.nih.gov/ij/, 1997–2018). Extracted data were treated using a developed in-house script in MATLAB (MathWorks). Briefly, data were normalized to baseline fluorescence (ΔF/F0), and each peak was analyzed for the following parameters: amplitude, rise time, maximal upstroke velocity, time to 50 and 80% decay, maximal decay velocity, and time between peaks, as previously described (Abecasis et al., 2019).

#### Cardiotoxic Drug Exposure

After 15 days of culture, microencapsulated cardiac microtissues and hiPSC–CM controls were harvested from suspension culture and transferred to 96-well plate (two to five capsules were inoculated per well). Cells were first evaluated for metabolic activity and cell viability as explained in section “*Cell Counting, Viability, and Metabolic Activity*.” Then, the cultures were washed twice with DPBS and incubated with doxorubicin or paclitaxel (0.1, 0.5, 1, 5, 10, 50, and 100 μM, MedChemExpress) for 72 h in a humidified incubator with 5% (vol/vol) CO_2_ in air at 37°C under agitation (90 rpm). After this period, cells were assessed again for metabolic activity and cell viability (see section “*Cell Counting, Viability, and Metabolic Activity”*).

#### Atomic Force Microscopy

Atomic force microscopy (AFM) characterization was performed to compare surface topography and mechanobiological behavior of alginate capsules and the cardiac microtissues 15 days after microencapsulation. Samples were collected and fixed as indicated previously (see section “*Whole Mount”*). Fixed samples were washed twice with DPBS and stored at 4°C until AFM characterization. Alginate microcapsules were embedded in 2% (wt/vol) high-melting-temperature agarose (Lonza), forming a thin film, which was kept hydrated up to AFM analysis. All measurements were performed in an Asylum Research MFP-3D Stand Alone system, whereas samples were immersed in PBS buffer. Surface topography measurements were acquired in alternate contact mode using commercially available AFM probes (Olympus BL-AC40-TS; *f*_0_ = 110 kHz; *k* = 0.09 N/m). Acquisition of load–unload force curves (force spectroscopy) was performed with the same probes, after previous calibration through thermal tuning (Green et al., 2004). Asylum Research’s analysis software packages loaded in IGOR Pro software (WaveMetrics) were used to generate low-order plane-fitted topography images and determine average pore diameter. Load–unload curves were analyzed in the same software, being considered curves from, at least, three capsules per condition. Apparent elastic moduli of the samples were determined by fitting the elastic Sneddon contact model for conical indenters (Sneddon, 1965) to the unload curves.

### Statistical Analysis

Statistical analysis was performed with GraphPad Prism6 (GraphPad Software Inc.). Values are represented as mean ± standard error of the mean (SEM) of measurements or assays in independent alginate microcapsules from one or two independent differentiation batches, as stated in the respective results figures. Statistical significance was determined by Student *t* test. *p* < 0.05 was considered as statistically significant.

## Results

### Establishment of a 3D hiPSC–Derived Cardiac Microtissue Model

In this work, we developed a 3D cardiac microtissue model using CM, EC, and MC derived from hiPSC microencapsulated in alginate. Differentiation of hiPSC into CM lineage was performed as previously described by our group (Correia et al., 2018), yielding 3D aggregates of hiPSC–CM with uniform size distribution (average size: 142 ± 26 μm) and high cell viability (Supplementary Figure 1A). Noteworthy, the hiPSC–CM aggregates showed high CM purity by the end of the differentiation protocol, with majority of the cells expressing SIRPα/β (81% ± 1%), VCAM-1 (73% ± 5%) and cTnT (77%) at day 15 of differentiation (Supplementary Figure 1B). In parallel, hiPSC were also differentiated into EC and MC, using a monolayer-based differentiation protocol developed by other authors (Giacomelli et al., 2017). This protocol generated a mixed population of cells expressing CD31 (27% ± 4%), VE-Cad (21% ± 3%), vimentin (90% ± 1%), and α-SMA (35% ± 12%), which are markers for EC (CD31 and VE-Cad) and fibroblasts and/or vascular smooth muscle cells (vimentin and α-SMA; hereafter designated as hiPSC–EC+MC, Supplementary Figure 1C). After 10 days of differentiation, hiPSC–EC+MC were harvested and co-cultured with hiPSC–CM aggregates, collected at day 15, within alginate microcapsules.

**FIGURE 1:**
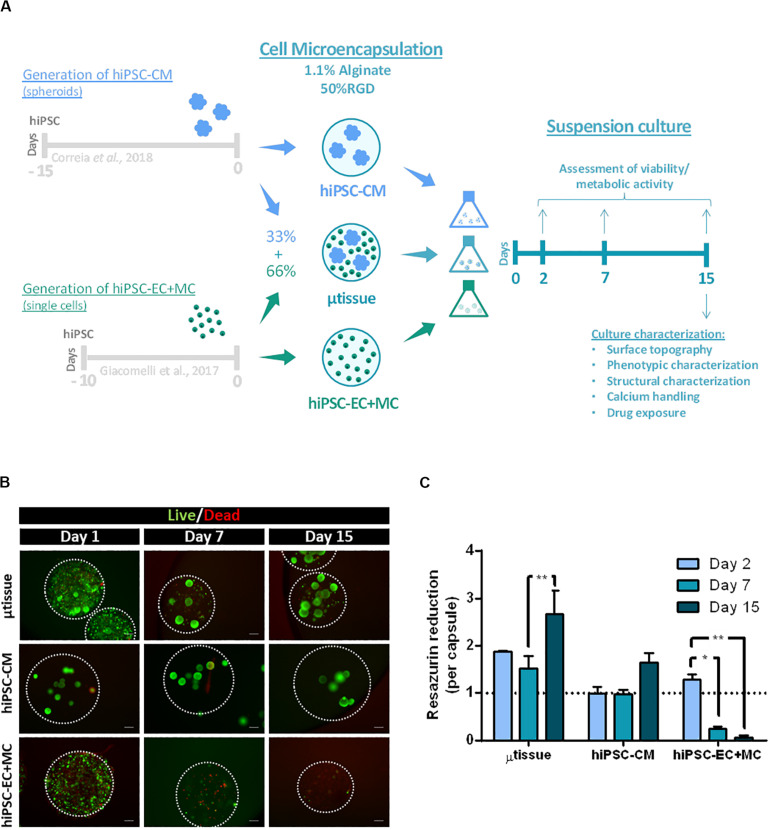
**(A)** Schematic representation of the alginate microencapsulation protocol for cardiac microtissue (μtissue) formation and respective control cultures hiPSC–CM and hiPSC–EC+MC. Capsules were maintained in dynamic suspension culture until day 15 post-microencapsulation for further microtissue characterization. **(B,C)** Evaluation of cell viability and metabolic activity for the cardiac microtissue (μtissue) and control cultures (hiPSC–CM and hiPSC–EC+MC). **(B)** Viability analysis of the three microencapsulated cell cultures throughout time (days 1, 7, and 15), stained with fluorescein diacetate (FDA—live cells, green) and propidium iodide (PI—dead cells, red). Microcapsules are marked with white dashed line. Scale bar: 200 μm. **(C)** Metabolic activity of the three microencapsulated cell cultures during time (days 2, 7, and 15), measured by the capacity of reduction of Presto-Blue^TM^ Viability Reagent, normalized by capsule and to hiPSC–CM culture condition on day 2. Data represented as mean ± SEM; *n* = 3 independent measurements from one differentiation batch; *p < 0.05, **p < 0.005.

Cell/aggregate microencapsulation in alginate (1:1 mixture of Ultrapure MVG alginate and NOVATACH MVG GRGDSP peptide-coupled alginate; see section “*3D hiPSC–Derived Cardiac Microtissue Culture”*) was carried out using the methodology already reported by our group (Rebelo et al., 2015, 2018; Estrada et al., 2016). A preliminary study was performed to evaluate the impact of aggregate concentration [hiPSC–CM aggregates/mL of 1.1% (wt/vol) alginate solution] on the percentage of empty capsules generated. Results showed that the percentage of empty capsules was reduced when using a concentration of at least 12,000 aggregates/mL of alginate (Supplementary Figure 1D), and thus this condition was selected to perform the microencapsulation of cell aggregates. For the generation of 3D microencapsulated cardiac microtissues, hiPSC–CM aggregates were mixed with hiPSC–EC+MC single cells in a 1:2 cellular ratio in order to obtain a cell composition similar to what has been reported for human adult heart: approximately 33% CM, 17% EC, and 50% MC (Bergmann et al., 2015). The cell/aggregate suspension was then microencapsulated in alginate mixture (hereafter designated as cardiac microtissues; Figure 1A). Microencapsulation of hiPSC–CM aggregates alone and hiPSC–EC+MC single cells was also performed and used as controls (Figure 1A). All cell microencapsulated conditions (cardiac microtissues, hiPSC–CM aggregates, and hiPSC–EC+MC) were cultured in agitated suspension conditions for additional 15 days.

Cell viability and metabolic activity of all cultures were evaluated after 1, 7, and 15 days of microencapsulation by live/dead staining and PrestoBlue^TM^ assay (Figures 1B,C). The results showed that cardiac microtissues maintained high cell viability throughout culture time, displaying significantly higher metabolic activities than both control cultures (Figures 1B,C; at day 15 μtissue vs. hiPSC–CM *p* = 0.0074 and μtissue vs. hiPSC–EC+MC *p* < 0.001). This result is also related with the higher number of cells inoculated within the cardiac microtissues microcapsules when compared to the values used in control cultures. Noteworthy, our data also show evidence of the protective effect of hiPSC–CM on hiPSC–EC+MC survival. In particular, we observed a significant drop in viability and metabolic activity from day 7 onward in hiPSC–EC+MC cultures, contrasting with cardiac microtissues and hiPSC–CM monocultures that maintained high cell viability and metabolic activity through time (Figures 1B,C). The significant increase in metabolic activity of cardiac microtissues from days 7 to 15 may indicate either an increase in cell numbers due to cell proliferation of non-myocyte cells or an increase in metabolic capacity of hiPSC–CM aggregates, as previously described by our group (Correia et al., 2018). In fact, a similar profile was observed in hiPSC–CM control culture that showed a peak in metabolic activity at day 15.

**FIGURE 2:**
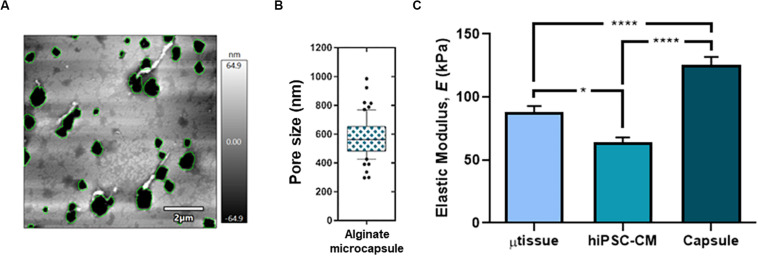
Topographical characterization of the cardiac microtissue and the alginate microcapsule at day 15 post-microencapsulation. Surface topography **(A)** and pore size (**B**; *n* = 61 independent measurements from two differentiation batches) of the alginate capsule by atomic force microscopy (AFM). **(C)** Mechanical characterization (elastic modulus, *E*) for the microtissue (μtissue; triculture), hiPSC–CM and the empty alginate capsule via AFM. Data represented as mean ± SEM; *n* > 40 independent measurements from two differentiation batches; *p < 0.05, ****p < 0.0001.

In addition, characterization of mechanical and topographical properties was performed via AFM in empty alginate capsules and encapsulated cultures (hiPSC–CM aggregates and cardiac microtissue). AFM topographical imaging evidenced an alginate porous surface structure (Figure 2A), with heterogeneity, particularly in the microcapsule constructs containing hiPSC–CM aggregates and cardiac microtissue when compared with cell-free capsules (data not shown), demonstrating cell–biomaterial interaction and its impact on morphology and topography. Additionally, analysis of the AFM images showed that the microcapsule average pore diameter was 579.6 ± 137.0 nm (Figures 2A,B), enabling effective diffusion of nutrients, metabolites, and drugs into the microcapsule. Furthermore, characterization was performed via load–unload force curves (force spectroscopy) for the empty alginate capsules and encapsulated at the last day of culture (Figure 2C). The calcium RGD-alginate microcapsule biomaterial alone denoted an elastic modulus of 132.3 ± 53.7 kPa, significantly superior to the cellularized constructs (Figure 2C). This seems to indicate that the microenvironment in the presence of the cells is less stiff, when compared to the biomaterial alone. In addition, in comparison to the encapsulated hiPSC–CM aggregates, the cardiac microtissue’s elastic modulus is significantly higher (Figure 2C), exhibiting a stiffer microenvironment more resistant to deformation, which may be consistent with a remodeling of the cardiac microenvironment resulting from the presence of the non-myocyte cells.

### Phenotypic, Structural, and Functional Characterization of the 3D hiPSC-Derived Cardiac Microtissue

**FIGURE 3:**
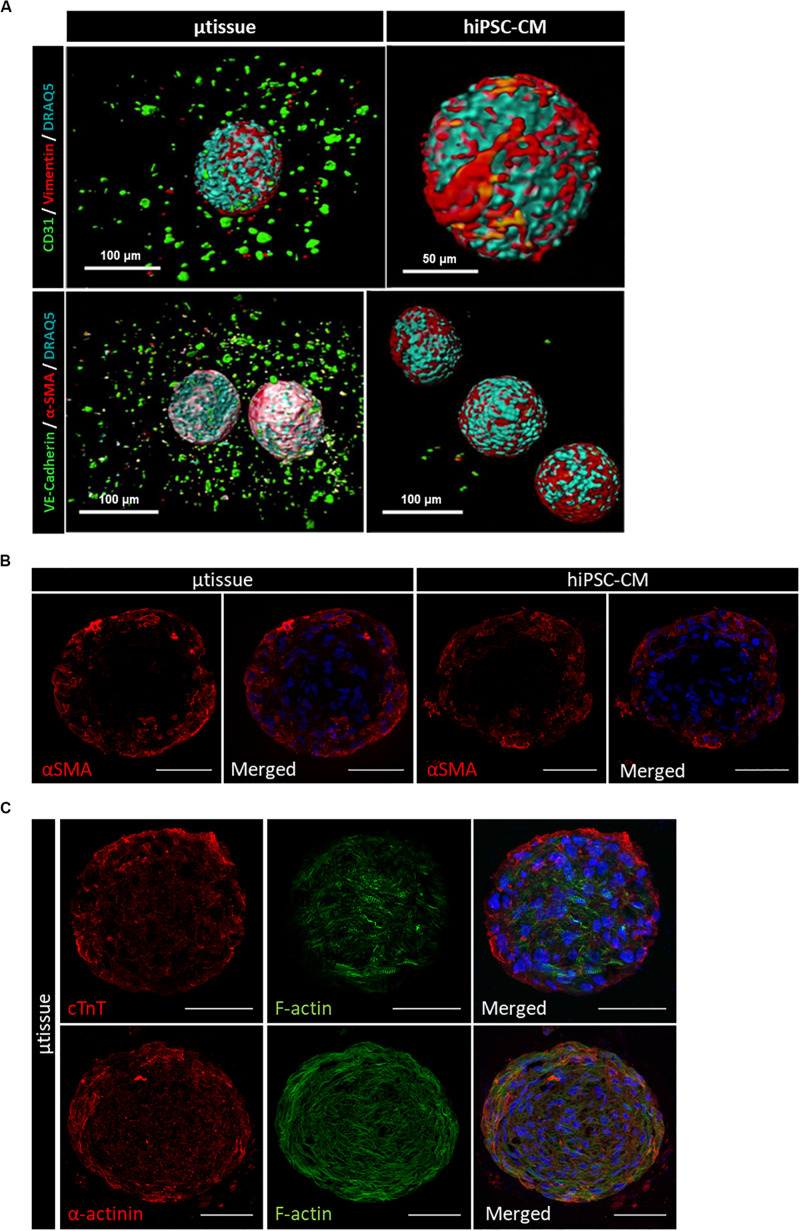
Phenotypic characterization of cardiac microtissue (μtissue) and hiPSC–CM control culture by immunofluorescence microscopy at day 15. **(A)** Light-sheet immunofluorescence microscopy of the whole-mount microencapsulated cardiac microtissue and hiPSC–CM. Cells were stained for endothelial cell markers (CD31 and VE-cadherin, green) and MC markers (vimentin and α-SMA, red). Images were counterstained with DRAQ5 (nuclei, cyan). Scale bar = 100 μm. **(B,C)** Immunofluorescence microscopy of cryosections of cardiac microtissue and hiPSC–CM aggregates. **(B)** Cells were stained for α-smooth muscle actin (α-SMA, red). Images were counterstained with and DAPI (nuclei, blue). Scale bar = 50 μm. **(C)** Cells from cardiac microtissue (μtissue) were stained for sarcomeric structural proteins (cTnT and α-actinin, red). Images were counterstained with phalloidin (f-actin, green) and DAPI (nuclei, blue). Scale bar = 50 μm.

**FIGURE 4:**
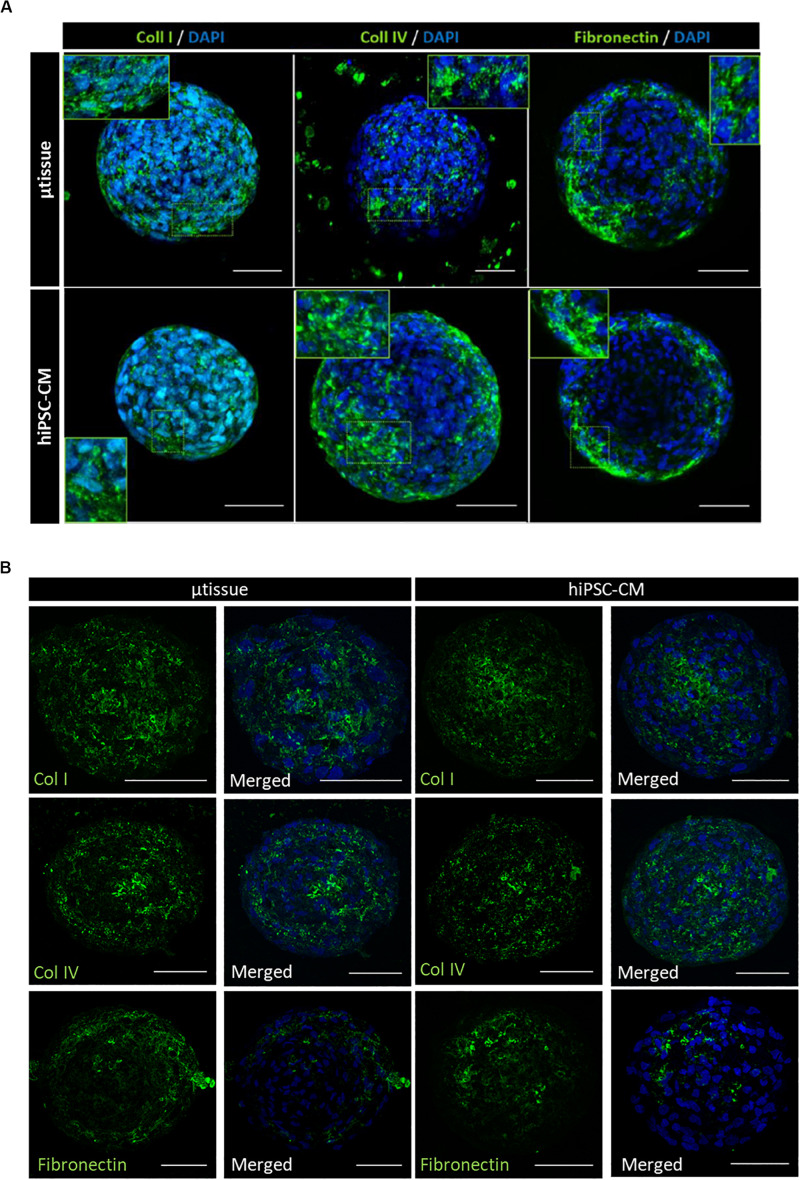
Phenotypic characterization of cardiac microtissue (μtissue) and hiPSC–CM by immunofluorescence microscopy after 15 days’ culture. Cells were stained for ECM proteins (collagen I, collagen IV, and fibronectin). Images are counterstained with DAPI. **(A)** Multiphoton immunofluorescence microscopy of the whole-mount microencapsulated cardiac microtissue and control hiPSC–CM. Scale bar = 50 μm. **(B)** Immunofluorescence microscopy of the cryosections of cardiac microtissue and control hiPSC–CM monoculture for characterization of the hiPSC–CM aggregates. Scale bar = 50 μm.

Cardiac microtissues were characterized at phenotypic, structural, and functional level and compared with hiPSC–CM. LSFM was used to confirm the presence of the non-myocyte cellular populations, namely, EC (stained for CD31 and VE-cadherin) and MC (stained for vimentin and α-SMA), as single cells within alginate microcapsules after 15 days of culture (Figure 3A). Vimentin and α-SMA staining was also observed within the aggregates of the cardiac microtissues either by LSFM (Figure 3A) and IFM of the cryosections (Figure 3B). These MC most likely originate from the percentage of cells in the CM differentiation protocol that do not express cardiac specific markers (Supplementary Figure 1B), because these stainings were also observed in the hiPSC–CM aggregates control culture (Figures 3A,B). Cardiac microtissues showed expression and organization of sarcomeric structural proteins (cTnT and α-actinin) after 15 days of culture (Figure 3C). In addition, f-actin counterstaining clearly illustrates assembly and organization of myofibrils (Figure 3C). Staining for collagen I, collagen IV, and fibronectin confirmed expression of these ECM components in cardiac microtissues by imaging through whole-mount multiphoton microscopy (Figure 4A) and cryosections’ confocal microscopy (Figure 4B). Noteworthy, collagen IV staining was more noticeable inside the cardiac microtissue alginate capsule (and outside the hiPSC–CM aggregates; Figure 4A), when compared to hiPSC–CM control, likely originated from the non-myocyte cells.

**FIGURE 5:**
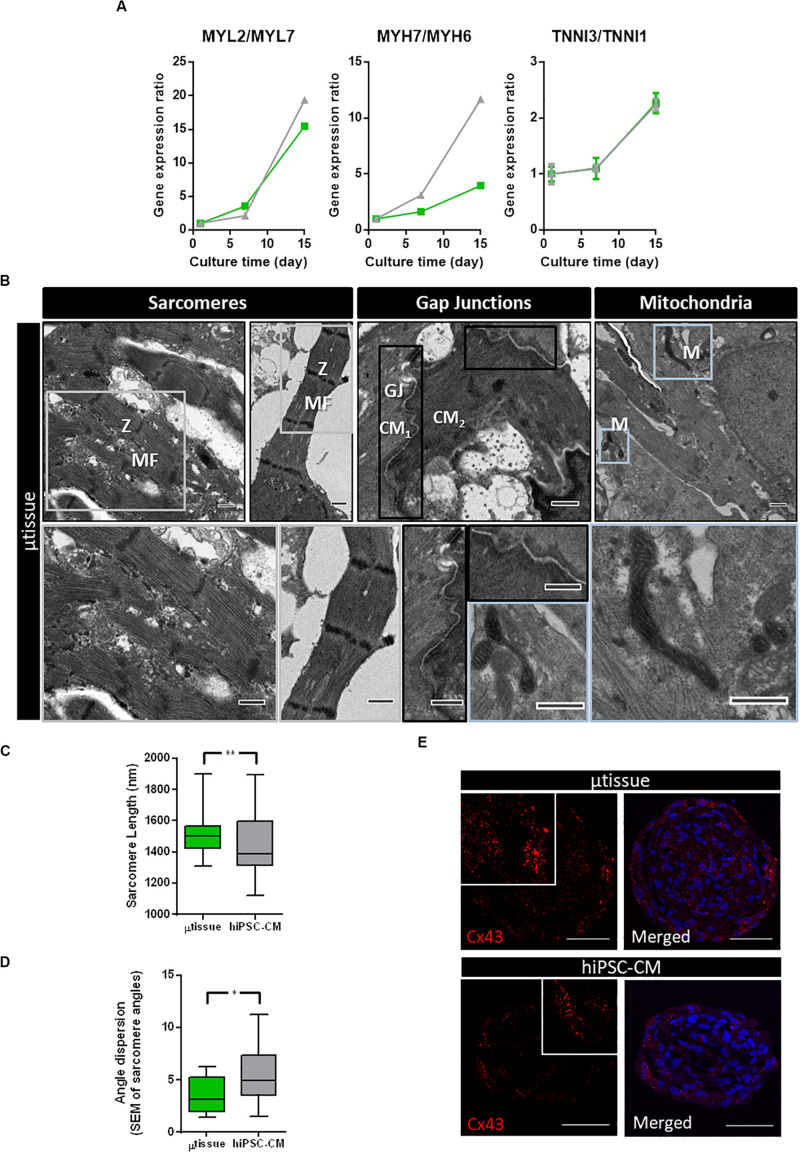
Characterization of structural maturation of the cardiac microtissues after 15 days of culture. **(A)** Analysis of gene expression by RT-qPCR of specific genes associated with cardiomyocyte structure and organization in cardiac microtissue (green) and hiPSC–CM (gray) throughout the culture time. Expression ratios for cardiac isoforms of mature (MYL2, MYH7, and TNNI3) and immature (MYL7, MYH6, and TNNI1) phenotypes were displayed. Gene expression was normalized to day 1. Data represented as mean ± SEM; *n* = 3 independent measurements from one differentiation batch. **(B)** Transmission electron microscopy images of cardiac microtissue (μtissue) and hiPSC–CM culture: (i) aligned myofibrils, composed by sarcomeres with organized Z-disks, A-bands, and I-bands; (ii) cell junctions (gap junctions, GJ) between adjacent hiPSC–CM; and (iii) abundant mitochondria (M). Scale bar: 2 μm. **(C)** Analysis of sarcomere length of cardiac microtissue (μtissue, green) and hiPSC–CM culture (gray). Data represented as mean ± SEM; *n* > 40 independent measurements from one differentiation batch; **p < 0.01. **(D)** Analysis of myofibril alignment (as shown by myofibril angle dispersion) of cardiac microtissue (μtissue, green) and hiPSC–CM control (gray). Data represented as mean ± SEM; *n* > 8 independent measurements from one differentiation batch; *p < 0.05. **(E)** Phenotypic characterization of cardiac microtissue (μtissue) and hiPSC–CM culture by immunofluorescence after 15 days’ culture. Cells were stained for connexin 43 (Cx43, red). Images are counterstained with DAPI (blue). Scale bar = 50 μm.

Gene expression analysis of cardiac microtissues by RT-qPCR revealed an increase in expression of gene isoforms associated with mature sarcomeric structures when compared to more immature isoforms (MYL2/MYL7, MYH7/MYH6, and TNNI3/TNNI1 ratios) along culture time (Figure 5A). Although similar profiles were observed in terms of sarcomeric gene expression for cardiac microtissue and hiPSC–CM, ultrastructural analysis showed differences in terms of sarcomeric length and alignment, indicative of hiPSC–CM maturation. Ultrastructure analyses of the cardiac microtissues show the presence of sarcomeres with organized *Z*-disks, A-bands, and I-bands by day 15 of culture (Figure 5B), which were not observed by the beginning of the culture (data not shown). Intercalated disks between adjacent hiPSC–CM and abundant mitochondria were also identified (Figure 5A). Analysis of sarcomere length and alignment from images obtained by transmission electron microscopy (TEM) suggested improved structural maturation of hiPSC–CM in the cardiac microtissues compared to the control hiPSC–CM aggregates. In particular, sarcomere length and myofibril alignment were significantly higher in cardiac microtissues when compared to hiPSC–CM aggregates (Figures 5C,D). In agreement, IFM of aggregate cryosections indicated evidences of increased expression of Cx43 in cardiac microtissues than in hiPSC–CM aggregates (Figure 5E), which could also be indicative of improved structural maturation of the formers, as previously described by other studies (Hussain et al., 2013; Giacomelli et al., 2020).

**FIGURE 6:**
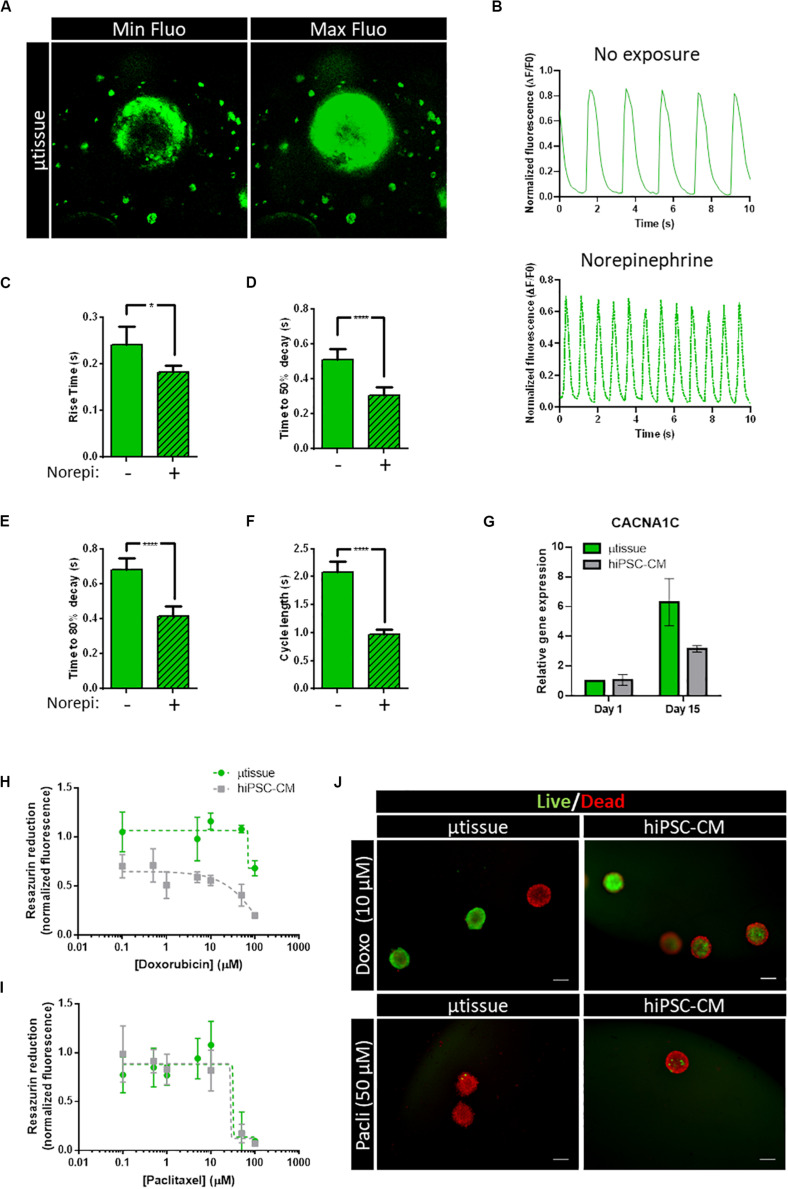
**(A–F)** Characterization of calcium handling kinetics of cardiac microtissue by fluorescent calcium imaging at day 15 post-microencapsulation. **(A)** Representative images of the maximum and minimum fluorescence of the videos analyzed. **(B)** Representative profile of spontaneous calcium transients in the cardiac microtissue: without drug exposure and with exposure to 60 μM of norepinephrine. **(C–F)** Calcium kinetics parameters obtained from the analysis of the transients: **(C)** rise time, **(D)** time to 50% decay, **(E)** time to 80% decay, and **(F)** cycle length. Data represented as mean ± SEM; *n* > 8 independent measurements from one differentiation batch; *p < 0.05, ****p < 0.0001. **(G)** Analysis of gene expression by RT-qPCR of voltage-dependent L-type calcium channel subunit α-1C (CACNA1C). Data represented as mean ± SEM; *n* = 3 independent measurements from one differentiation batch. **(H–J)** Evaluation of metabolic activity and cell viability of the cardiac microtissue (μtissue) after 72-h exposure to doxorubicin or paclitaxel. **(H,I)** Dose–response curve showing the effect of **(H)** doxorubicin and **(I)** paclitaxel on cell metabolic activity measured by the capacity of reduction of Presto-Blue^TM^ Viability Reagent. Values were normalized to metabolic activity of cardiac microtissue before exposure. Data represented as mean ± SEM; *n* = 3 independent measurements from one differentiation batch. **(J)** Representative images of cell viability analysis of cardiac microtissues and hiPSC–CM after 72-h exposure to 10 μM of doxorubicin (Doxo) and 50 μM of paclitaxel (Pacli), stained with fluorescein diacetate (FDA—live cells, green) and propidium iodide (PI—dead cells, red). Scale bar: 200 μm.

Calcium imaging was performed using a fluorescent calcium indicator (Figures 6A–F, Supplementary Figure 2 and Supplementary Video 1), enabling the evaluation of the functionality of generated cardiac microtissues and estimation of different calcium kinetics’ parameters. Spontaneous calcium transients were observed in the cardiac microtissues, indicating the presence of functional calcium handling machinery (Figure 6B). Exposure to an adrenergic agonist (norepinephrine, 60 μM) resulted in a positive chronotropic response (Figure 6B). Analysis of the calcium kinetics’ parameters revealed that exposure to norepinephrine resulted in decrease in rise and decay time (Figures 6C–F) and decrease in cycle length (Figure 6G), demonstrating functional β-adrenergic response. Moreover, increased expression of the L-type calcium-channel subunit α-1C (CACNA1C) was also observed over time in the cardiac microtissue (Figure 6H), which could be indicative of increased calcium handling functionality in the cardiac microtissue.

To evaluate the feasibility of this cardiac microtissue for application in toxicology, a proof-of-concept study was performed by exposing cell microcapsules to two chemotherapy anticancer drugs with known cardiotoxicity effects (Figures 6H–J): doxorubicin and paclitaxel (Bovelli et al., 2010). Our results indicate that cardiac microtissues were sensitive to both drugs. A decrease in cell viability and metabolic activity after exposure to doxorubicin and paclitaxel in a concentration-dependent manner was observed (Figures 6H,I). Noteworthy, hiPSC–CM seem to have an increased sensitivity to doxorubicin when compared to the cardiac microtissue showing lower metabolic activities (Figure 6H). No major differences in sensitivity were observed between hiPSC–CM and the cardiac microtissue for the paclitaxel exposure (Figure 6I), both displaying low metabolic activities for concentrations greater than 10 μM. Representative images of live/dead staining after exposure to doxorubicin and paclitaxel are shown in Figure 6J.

## Discussion

Drug development is a long and costly process with low success rates, in which cardiotoxicity is one of the major reasons for failure (Eder et al., 2016). Current preclinical testing relies mainly on (i) *in vitro* systems that have low compliance with high-throughput screenings; or (ii) cost- and labor-intensive animal models that differ from human physiology and should be restricted to a minimum use from an ethical point of view (Eder et al., 2016). The development of biologically relevant and reliable human *in vitro* cardiac tissue models using hiPSC derivatives may circumvent some of these limitations, allowing better understanding of human cardiac diseases and improvement of the assessment of cardiotoxicity of new drugs in a human setting. In this study, we developed a novel *in vitro* 3D human cardiac microtissue by combining hiPSC–CM aggregates and hiPSC–EC+MC single cells inside alginate microcapsules.

Cell microencapsulation technology has been thoroughly explored by our group for different applications in (stem) cell bioprocessing, such as expansion/cryopreservation of hESC (Serra et al., 2011), culture of hepatocyte spheroids (Tostões et al., 2011; Rebelo et al., 2015), and tumor *in vitro* modeling (Estrada et al., 2016; Rebelo et al., 2018), showing positive impact on cell viability, phenotype and functionality. The small spherical capsules (approximately 1,500 μm in diameter) confer protection to shear stress, while creating an extracellular environment that enables diffusion of nutrients and soluble factors through the hydrogel structure and prevents the washout of ECM and soluble factors (Tostões et al., 2011; Serra et al., 2012; Rebelo et al., 2015, 2018; Estrada et al., 2016); therefore, a microenvironment is generated in which the effect of paracrine interactions between the different cardiac cell types (hiPSC–CM and hiPSC–EC+MC) can be studied. The pore size estimated by AFM (579.6 ± 137.0 nm) seem to support that appropriate diffusion of nutrients and oxygen has occurred, as well as the proper diffusion of the cardiotoxic drugs in the proof-of-concept toxicology study. Additionally, alginate is biomaterial with no animal-derived components, and the combination of AggreWell technology and alginate microencapsulation is compatible with agitated suspension cultures (e.g., shake flasks and bioreactors) and high-throughput screening studies. Furthermore, if required, separation of both cell types (aggregates and single cells) is facilitated by the end of the co-culture period, enabling the study and characterization (e.g., flow cytometry, mass spectrometry, and functional characterization, etc.) of each population individually without the need of adding extra cell dissociation steps that usually compromise cells’ viability and quality attributes.

Another main benefit of cell microencapsulation technology is the possibility of designing the scaffold environment with specific biomaterials and/or hydrogels that can be further functionalized to create tailored microenvironments that regulate cell fate decisions, i.e., improved cell viability, expansion/differentiation, and/or functionality. In this study, a mixture of peptide-coupled alginate containing RGD motifs (arginine–glycine–aspartic acid) was used to promote attachment and survival of anchorage-dependent single cells (hiPSC–EC+MC), as previously described for *in vitro* cardiac cell-based models (Shachar et al., 2011). Our designed hydrogel showed to be suitable for the preservation of cell viability and phenotype of all cardiac populations within 15 days of culture. Noteworthy, we showed for the first time evidences of the protective effect of hiPSC–CM on hiPSC–EC+MC survival, similarly to what has been described for *in vivo* cardiac development conditions (Hsieh et al., 2006). Our findings may be related with paracrine factors produced by CM known to be responsible for maintenance of non-myocyte cells viability during *in vivo* cardiac development, namely, VEGF-A, or angiopoietin (Hsieh et al., 2006). In fact, a previous study from our group has identified expression of angiogenic factors (including VEGF) in the secretome hiPSC–CM aggregates (Sebastião et al., 2020). A deeper understanding of how hiPSC–CM communicate with hiPSC–EC+MC will be important not only to develop more physiologically relevant cardiac tissue models but also to design advanced therapies for cardiac regeneration.

The use of a combination of different bioimaging techniques confirmed the organization of sarcomeric structure in hiPSC–CM aggregates as well as the presence of the non-myocyte cellular populations within the alginate capsule of cardiac microtissues at day 15 of culture. Noteworthy, gene expression analysis and ultrastructure characterization of the cardiac microtissues showed that hiPSC–CM displayed improved structural maturation with culture time. This result is aligned with other studies reporting the importance of EC and CF in enhancing the maturation of hiPSC–CM at the structural, functional, and metabolic level. In a recent work, Giacomelli and coworkers identified key mechanisms in the tricellular interactions, involving cAMP/β-adrenergic and cell junction assembly pathways (Giacomelli et al., 2020). In fact, the microencapsulated cardiac microtissues developed herein also showed increased expression of the gap junction protein Cx43.

Other heterotypic communication mechanisms, such as cell–ECM interactions or paracrine effects, have also been shown to impact on the maturation of hiPSC–CM (Fong et al., 2016; Abecasis et al., 2019). Indeed, the alginate microencapsulation supported the remodeling of the cells’ microenvironment, namely, the deposition of ECM components, such as collagen IV. However, the observed ECM deposition may have impacted the hiPSC–CM maturation not only directly through cell–ECM communication but also through the changes in the physical properties of the supporting biomaterial. The results from AFM indicated an increase in the elastic modulus when comparing the cardiac microtissue with the hiPSC–CM; which may be related to the observed ECM deposition, concordantly to previous studies with multicellular cardiac patches (Stevens et al., 2009). Overall, the elastic modulus estimated for the cardiac microtissue is within the range of values reported for the human cardiac muscle (10–15 kPa; Mathur et al., 2016; Kofron and Mende, 2017; Dunn and Palecek, 2018). Further optimization of this cardiac microtissue can be evaluated by extending the time in culture to increase microtissue maturation or by improving the complexity/functionalization of the biomaterial used for microencapsulation (Dalheim et al., 2019), as previously studied in other cellular models (Almeida et al., 2017). In the future, a time-resolved analysis of the topographical and physical changes of the cardiac microtissue would be beneficial for the characterization of the profiles along time and identification of the culture timepoint where a better recapitulation of the human cardiac microenvironment would occur.

The microencapsulated hiPSC–based cardiac microtissues displayed functional calcium signaling, as well as positive response to known cardiotoxins. A recent study using 3D hiPSC–derived cardiac microtissues has reported an IC_50_ 16 μM for doxorubicin (Archer et al., 2018), which is the same magnitude but lower when compared to our results for the hiPSC–CM control (approximately 56 μM). More importantly, our results show that there seems to be a significant difference in the doxorubicin resistance comparing the hiPSC–CM and the cardiac microtissue, because the cardiac microtissue viability was maintained up to 50 μM and only demonstrated a drop at 100 μM (not reaching the IC_50_). This result might suggest that the co-culture of the different cell types in this configuration contributed to drug resistance, to some extent, although further testing should be performed with additional hiPSC lines. Indeed, a study from Burridge and coauthors showed that patient-specific hiPSC–CM can recapitulate the predilection to doxorubicin-induced cardiotoxicity of individual patients at the cellular level (Burridge et al., 2016), strengthening the importance of genetically diverse hiPSC–derivatives for cardiotoxic studies in future studies. In contrast, the same difference was not observed in paclitaxel exposure, indicating a drug-dependent response. A study from the same group has also generated a “cardiac safety index” to reflect the cardiotoxicities of existing tyrosine kinase inhibitors, using individual populations of hiPSC–CM, hiPSC–EC, and hiPSC–CF (Sharma et al., 2017). Our designed cardiac microtissue may be used to complement these toxicological screenings, enabling the inclusion of the effect of paracrine interaction between the different cellular compartments and may also be applied for pathophysiological studies, similarly to recently published reports incorporating diseased cells (Giacomelli et al., 2020).

In this study, we demonstrated the application of alginate microencapsulation technology for cardiac tissue modeling where the cardiac cell populations were generated using protocols that were reported to ensure high differentiation efficiency and robustness across different hiPSC lines (Giacomelli et al., 2017; Correia et al., 2018). Although we performed two independent differentiation runs with one hiPSC line, additional studies with replicate differentiation batches of multiple hiPSC lines are needed to validate the robustness of the cardiac microtissue model. We believe that this very flexible system can be combined with several other reported hiPSC–CM maturation techniques, such as medium supplementation or biomaterial functionalization (or even by using starting material, which is already in advanced state of maturation) to achieve higher hiPSC–CM functional maturation and recapitulation of cardiac microenvironment. Because our microtissue model focused mainly on the paracrine interaction between the different cardiac cell populations, further studies are needed to understand if direct cell–cell communication would have an impact in the phenotypic and functional features of the cardiac microtissue model. In particular, the extension of the culture time, functionalization of the alginate microcapsule, and/or screening of different hiPSC lines might be evaluated and optimized to promote cell migration and physical interaction between CM and stromal cell. Nevertheless, this study is a step forward on the development of a biologically relevant hiPSC–derived cardiac microtissue that recapitulates features of the human cardiac microenvironment and is compliant with the larger numbers needed for pharmacological screenings.

## Data Availability Statement

The raw data supporting the conclusions of this article will be made available by the authors, without undue reservation.

## Author Contributions

BA, MS, and PA: conception and design. BA, PC, HA, and TC: experimental work. All authors contributed to data analysis and interpretation. BA and HA: manuscript writing. All authors contributed to manuscript revision. MS and PA coordinated the study and approved the final manuscript. All authors contributed to the article and approved the submitted version.

## Conflict of Interest

The authors declare that the research was conducted in the absence of any commercial or financial relationships that could be construed as a potential conflict of interest.
