# Supplementary material for: Toward a Microencapsulated 3D hiPSC-Derived in vitro Cardiac Microtissue for Recapitulation of Human Heart Microenvironment Features
Source: Front Bioeng Biotechnol. 2020 Nov 5;8:580744. doi: 10.3389/fbioe.2020.580744 (PMC7674657; doi:10.3389/fbioe.2020.580744)
Supplement: Supplementary file 1 [file Table_1.docx]

Supplementary Material

Supplementary Figure 1. (A-B) Phenotypic characterization of hiPSC-CM aggregate after differentiation. (A) Fluorescence images of hiPSC-CM aggregates before microencapsulation. Viability analysis of hiPSC-CM aggregates stained with fluorescein diacetate (FDA-live cells, green) and propidium iodide (PI-dead cells, red). Scale bar: 200 μm. (B) Percentage of cells positive for the cardiac specific markers: SIRPα/β, VCAM-1 and cTnT determined by flow cytometry, at day 15 of hiPSC-CM differentiation. Data represented as Mean±SEM; n=2 independent measurements from two differentiation batches. (C) Characterization of hiPSC-EC+MC after differentiation. Percentage of cells positive for the endothelial specific markers CD31 and VE-Cad and for the mesenchymal markers Vimentin and α-SMA, determined by flow cytometry at day 10 of hiPSC-EC-FB differentiation. Data represented as Mean±SEM; n=3 independent measurements from three differentiation batches. (D) Percentage of empty capsules for different hiPSC-CM aggregate concentrations in alginate solution.

Supplementary Figure 2. Representative profile of spontaneous calcium transients obtained by fluorescent calcium imaging in hiPSC-CM at day 15 post-microencapsulation: without drug exposure and with exposure to 60 µM of norepinephrine.
